# Trauma, Stress, and Mental Health Outcomes

**DOI:** 10.26502/jppd.2572-519X0260

**Published:** 2025-09-20

**Authors:** Mo’men Bany-Mohammed, Syed Asim, Majid Elalami, Devendra K. Agrawal

**Affiliations:** Department of Translational Research, College of Osteopathic Medicine of the Pacific, Western University of Health Sciences, Pomona, California 91766 USA

**Keywords:** Anxiety, Behavioral therapy, Cognitive therapy, Depression, Epigenetics, Personality disorder, Personalized care, Post traumatic stress disorder, Psychiatric disorders, Stress, Stressors, Substance abuse, Trauma

## Abstract

Trauma and chronic stress represent critical and growing challenges in mental health across the lifespan, contributing to a wide spectrum of psychiatric conditions. These include post-traumatic stress disorder, depression, anxiety, substance abuse disorders, and personality disorders. While acute traumatic events trigger immediate psychological responses, long-term exposure to stressors can result in chronic biological and emotional impacts. These stressors interact with various neurobiological systems involving the hypothalamic-pituitary-adrenal axis, amygdala, hippocampus, and prefrontal cortex, leading to changes in emotional regulation, memory, and arousal. Epigenetic mechanisms and gene-environment interactions further contribute to individual vulnerability and the intergenerational transmission of risk. Clinically, trauma-related disorders often involve overlapping symptoms or co-occurring conditions, making diagnosis difficult and leading researchers to consider dimensional diagnostic models. Social support, resilience, and contextual factors such as access to care and socioeconomic status strongly influence outcomes, highlighting the importance of community-based and culturally informed interventions. Public health approaches emphasize trauma screening, early intervention, and multi-level prevention strategies. Evidence-based treatments such as trauma-focused cognitive behavioral therapy and pharmacologic options can be effective, particularly when adapted to individual and cultural needs. Emerging research on biomarkers, digital interventions, and personalized care holds promise but requires further validation. Addressing trauma and stress as public health priorities necessitates an integrated framework that combines clinical care, social policy, and structural equity. Future research should prioritize longitudinal studies to close existing gaps and inform scalable interventions. By advancing a trauma-informed lens across systems, it is possible to alleviate long-term psychological harm and promote resilience at both individual and societal levels.

## 1. Introduction

Trauma and stress are now widely recognized as key contributors to mental health outcomes. Trauma typically refers to events that involve actual or threatened death, serious injury, or sexual violence. These events may be experienced directly, witnessed in person, or learned about when they happen to close others [1]. In contrast, stressors are broader and can include any life events or ongoing circumstances that strain a person’s ability to cope. These stressors may be acute, such as losing a job, or chronic, such as living in poverty or managing a long-term illness [2]. While trauma is often sudden and extreme, stressors can be subtle and chronic; both can significantly impact psychological well-being. It is important to distinguish between trauma and stressors because each can influence mental health in different ways. Acute trauma is more often linked to intense, immediate reactions such as post-traumatic stress disorder (PTSD), whereas chronic stress is associated with slower, cumulative effects that can lead to depression, anxiety, and burnout [3]. However, researchers increasingly view trauma and stress as existing on a spectrum rather than as separate categories. Many individuals experience both types of adversity, and the combined burden is often associated with worse outcomes and greater functional impairment [4]. Exposure to trauma is common across the globe. It is estimated that more than 70 percent of people worldwide have experienced at least one traumatic event in their lifetime [5]. In certain groups, such as refugees, survivors of interpersonal violence, or people living in areas affected by armed conflict, these numbers are even higher. Chronic stressors are also widespread. People from a wide range of backgrounds report experiencing stress related to finances, caregiving, discrimination, or unstable housing. These forms of adversity have been independently linked to an increased risk for depression, substance use, and other psychological problems [6]. From a public health perspective, the mental health effects of trauma and stress are becoming more alarming. Modern life presents growing threats including climate-related disasters, political instability, and economic inequality, all of which contribute to the stress burden in populations. These rising exposures suggest that the incidence of trauma- and stress-related disorders may continue to increase, with serious consequences for individuals, families, and healthcare systems [7]. This paper will examine the relationship between trauma, stressors, and mental health by reviewing evidence from epidemiology, neuroscience, clinical research, and public health. By exploring both short-term and long-term effects, as well as individual and environmental factors that influence outcomes, the goal is to provide a well-rounded view of how adverse experiences shape mental health and what strategies can reduce their impact.

## 2. Epidemiology of Trauma and Stressors

Understanding the epidemiology of trauma and stress is essential for recognizing their impact on mental health at both individual and societal levels. Research has consistently shown that trauma and stressor exposure are widespread, though prevalence varies depending on age, gender, region, and socioeconomic status. Estimates suggest that more than half of adults in the general population report at least one traumatic event in their lifetime, with some studies placing this number even higher [8]. These events include a range of experiences such as physical or sexual assault, serious accidents, natural disasters, and exposure to violence or warfare. In addition to traumatic events, many individuals are exposed to chronic or repeated stressors. These may include financial insecurity, food instability, caregiving responsibilities, job strain, discrimination, and unsafe living environments. Unlike acute trauma, which tends to be sudden and clearly identifiable, chronic stressors often accumulate gradually over time and may not be recognized as mental health threats until symptoms develop [9]. However, both acute and chronic exposures can cause significant psychological harm.

Certain types of traumas carry a particularly high risk for mental health problems. For example, interpersonal violence, including childhood abuse, intimate partner violence, and sexual assault, is among the most strongly associated with the development of post-traumatic stress disorder and other conditions [10]. Trauma that involves betrayal or occurs in close relationships can lead to more complex psychological outcomes and difficulties with trust, attachment, and identity [11]. Mass trauma, such as that seen in natural disasters or armed conflict, has been linked to elevated rates of depression, anxiety, and grief in entire communities [12]. Some populations are disproportionately affected by trauma and stress. Refugees and displaced persons, for instance, often face both past trauma and ongoing stress related to housing, employment, and legal status [13]. People in low-income neighborhoods may live with chronic exposure to violence, housing instability, and food insecurity. Children and adolescents are also highly vulnerable, as exposure to trauma during critical developmental periods can have long-term consequences for emotional regulation and cognitive function [14]. When looking at prevalence across demographics, research has found that women are more likely to experience interpersonal trauma, while men are more likely to be exposed to accidents, physical assaults, and combat-related trauma [15]. Socioeconomic disadvantage, minority status, and lack of access to healthcare also increase both the risk of exposure and the severity of outcomes. These social and structural inequities contribute to persistent disparities in mental health across populations.

Chronic stressors are not always included in large epidemiological surveys, but newer research increasingly acknowledges their importance. For example, daily hassles such as workplace stress, caregiving duties, and financial strain have been associated with higher risks of depression and anxiety, especially when persistent or combined with traumatic history [16]. The concept of “allostatic load”, which is the cumulative physiological burden of chronic stress, has gained attention as a framework to understand how repeated stress contributes to long-term health outcomes [17]. Furthermore, trauma and stress exposures often co-occur. Someone who experiences childhood abuse may also face discrimination, poverty, or unstable housing later in life. This clustering of adverse experiences increases the risk for multiple forms of pathology, including PTSD, depression, substance use, and suicidality [18]. Research has also shown that the number of different types of traumatic or stressful events, rather than a single exposure, is a stronger predictor of negative outcomes. This is known as the “dose-response” relationship in trauma research [19]. Epidemiological studies serve as a foundation for understanding the burden of trauma and stress-related conditions. They guide the allocation of mental health services, help identify at-risk groups, and support public health efforts aimed at prevention and early intervention. While no population is completely immune, the effects of trauma and stress are not evenly distributed. Addressing this imbalance is essential for equitable care.

## 3. Pathophysiology and Mechanisms

**Figure 1:**
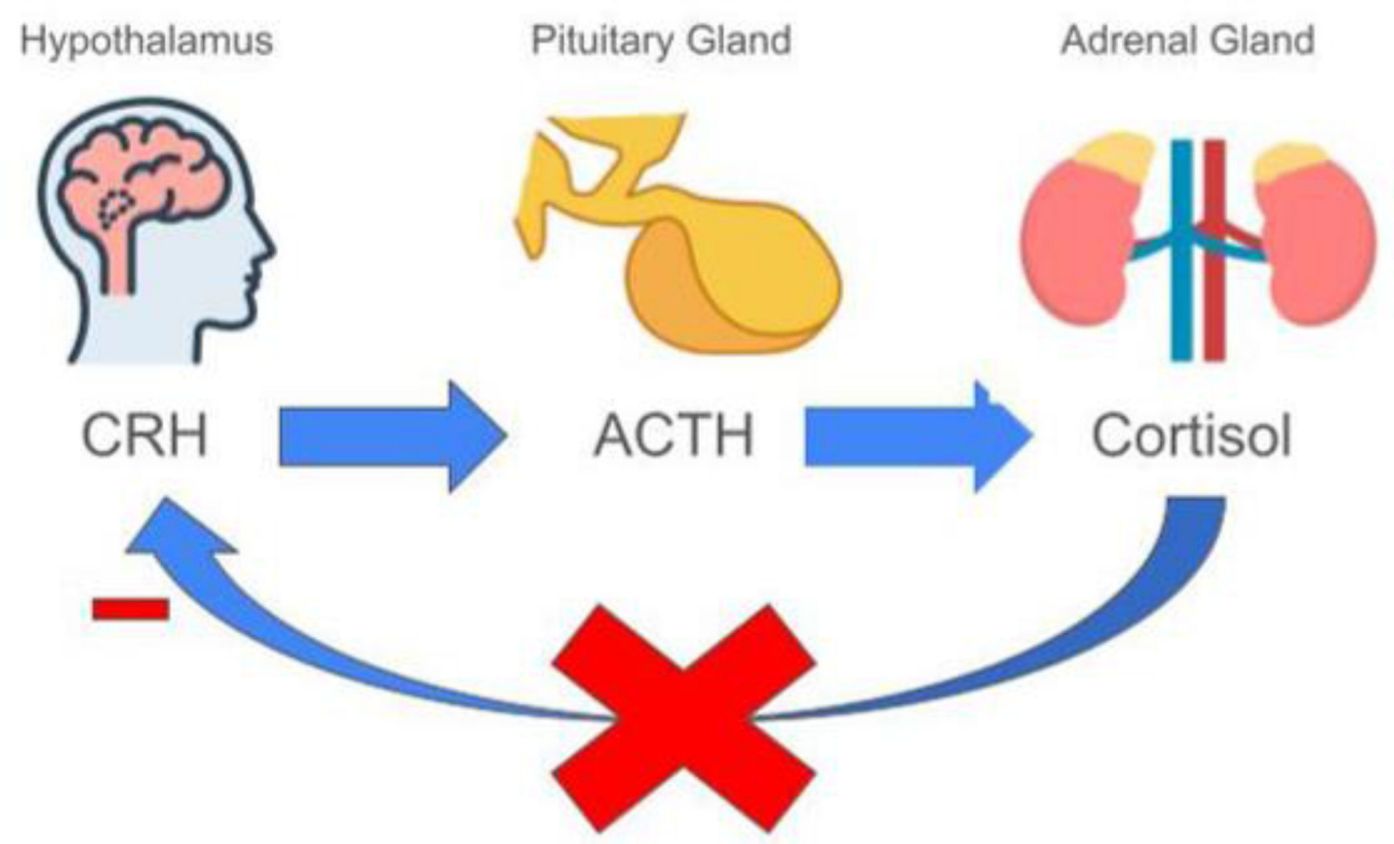
The effect of chronic stress on HPA axis.

The biological mechanisms underlying the mental health effects of trauma and stress are complex and multifactorial. Research has identified several key pathways through which adverse experiences can become biologically ingrained and influence long-term psychological functioning. These include neuroendocrine changes, structural and functional brain alterations, immune dysregulation, and gene-environment interactions. Many of these processes begin shortly after exposure and may persist for years, shaping an individual’s vulnerability or resilience to mental illness. One of the most widely studied systems affected by stress is the hypothalamic-pituitary-adrenal (HPA) axis, which regulates the body’s response to stress through the release of cortisol and other glucocorticoids (Figure 1). Traumatic experiences have been shown to dysregulate this system, leading to either hyperactivation or blunted responses depending on the timing, type, and chronicity of stress [20]. These alterations are thought to contribute to mood disorders, anxiety, and PTSD by disrupting the body’s ability to return to homeostasis after stress [21]. Changes in brain structure and function have also been observed in individuals exposed to trauma. Neuroimaging studies consistently show reduced volume in areas such as the hippocampus, which is critical for memory and emotion regulation, as well as functional changes in the amygdala and prefrontal cortex [22]. The amygdala often becomes hyperactive in response to perceived threat while the prefrontal cortex may become underactive [23]. These patterns are particularly evident in individuals with PTSD and may explain symptoms such as hypervigilance, intrusive memories, and emotional numbing [24].

Early life stress appears to have particularly strong and long-lasting effects on neurodevelopment. Adverse childhood experiences (ACEs) have been linked to structural brain changes, altered stress responses, and disrupted attachment systems. The concept of “biological embedding” refers to the idea that early experiences become encoded in physiological systems during sensitive developmental windows, potentially altering a person’s trajectory of health and behavior [25]. Epigenetic mechanisms have been proposed as a biological explanation for how environmental exposures can affect gene expression. Trauma has been associated with epigenetic changes in genes related to the stress response, such as those encoding glucocorticoid receptors [26]. These modifications can be stable over time and may be transmitted intergenerationally, raising the possibility that trauma exposure may have biological consequences for future generations. In addition to individual biology, the interaction between genes and environment is increasingly recognized as important. Certain genetic variants may alter sensitivity or resistance to trauma. For example, polymorphisms in genes related to serotonin transport or the HPA axis have been found to moderate the relationship between trauma exposure and psychiatric outcomes [27]. This suggests that risk for mental illness after trauma is not solely determined by the environment, but also by genetic predisposition and their interaction.

Another line of research has explored the role of inflammation and immune activation in trauma-related disorders. Individuals with PTSD and depression frequently show elevated levels of pro-inflammatory markers, such as interleukin-6 (IL-6) and C-reactive protein (CRP) [28]. Chronic stress can alter immune function, promoting low-grade inflammation that may contribute to fatigue, cognitive dysfunction, and mood disturbances. This has led to interest in anti-inflammatory treatments for certain trauma-related conditions. Trauma and stress also influence neuroplasticity, or the brain’s ability to adapt and change in response to experience. While neuroplasticity is essential for learning and recovery, chronic stress can impair the brain’s plasticity and ability to regulate emotional responses effectively. This is partly mediated by an excess in stress hormones, which can be neurotoxic particularly in areas like the hippocampus [29]. Sleep disturbances are another pathway through which trauma can influence mental health. Sleep problems are among the most common symptoms reported after trauma and are associated with worse outcomes. Disrupted sleep impairs memory processing, emotional regulation, and immune function, potentially reinforcing trauma-related symptoms [30]. There is also growing evidence that the gut-brain axis may play a role in stress-related disorders. Chronic stress and trauma have been associated with changes in the gut microbiota, which in turn can influence mood and behavior through neural, hormonal, and immune pathways [31]. While this area is still emerging, it offers new avenues for understanding how lifestyle and environmental factors might affect psychological resilience. These mechanisms often interact and amplify one another. For example, HPA axis dysregulation can lead to both immune changes and epigenetic alterations. Similarly, early life stress may sensitize individuals to later stress exposure, creating a “stress sensitization” effect that increases vulnerability over time [32]. This cumulative impact helps explain why some individuals develop chronic mental health problems after trauma, while others recover or remain resilient. Importantly, not all responses to trauma are pathological. Some individuals demonstrate remarkable resilience, which may be supported by genetic, neurobiological, and environmental protective factors. Understanding these mechanisms not only helps explain the development of mental illness but also points toward strategies for prevention and intervention.

Research into biomarkers of trauma exposure and recovery is ongoing. The goal is to identify measurable indicators that can help diagnose trauma-related conditions, predict treatment response, or monitor recovery over time. These may include cortisol levels, inflammatory markers, brain imaging findings, or epigenetic signatures [33]. However, there is not yet a single biomarker or biological test that can reliably diagnose any trauma-related condition, and current approaches remain largely clinical. Overall, the pathophysiology of trauma and stress involves multiple interacting systems. Advances in neuroscience, genetics, and immunology continue to shed light on how adverse experiences contribute to the development of mental health disorders. A better understanding of these mechanisms is essential for improving diagnosis, treatment, and prevention [34].

## 4. Clinical Manifestations

Trauma and chronic stress can result in a wide range of psychiatric conditions, with significant variability depending on the nature, duration, and timing of exposure. While PTSD is a well-known trauma-related diagnosis, it is far from the only clinical outcome. Many individuals develop other primary or comorbid mental health disorders or exhibit subthreshold symptoms that still cause significant impairment. PTSD is a psychiatric condition that occurs following exposure to a traumatic event. Its hallmark symptoms include intrusive memories or flashbacks, avoidance of reminders, negative changes in mood or cognition, and hyperarousal [35]. PTSD may develop immediately or with a delayed onset, and its severity can vary widely across individuals. Notably, not all trauma survivors develop PTSD. Estimates suggest that only a subset of exposed individuals, often around 10 to 20 percent, meet full diagnostic criteria of PTSD, though many more experience distressing symptoms [36].

Complex PTSD (C-PTSD) is a related but distinct diagnosis proposed to capture the effects of repeated or prolonged interpersonal trauma, such as childhood abuse or captivity. In addition to core PTSD symptoms, individuals with C-PTSD may experience emotional dysregulation, negative self-concept, and interpersonal difficulties [37]. This diagnosis is now recognized in the International Classification of Diseases (ICD-11) and is especially relevant in cases of chronic abuse or neglect [38]. Prolonged grief disorder has also gained attention in recent years. It involves persistent grief and functional impairment beyond what is expected in a typical bereavement process. It is now recognized in DSM-5-TR as a distinct disorder, often seen following the traumatic or unexpected death of a loved one [39]. Grief-related disorders may overlap with PTSD or depression, particularly when the loss is sudden or violent.

Adjustment disorder represents a more time-limited reaction to identifiable stressors. It is characterized by emotional or behavioral symptoms that arise within three months of a stressor and cause more distress or dysfunction than would typically be expected [40]. Though often considered a milder diagnosis, adjustment disorders can evolve into more chronic conditions if unrecognized or untreated.

Beyond trauma-specific diagnoses, exposure to adversity is also strongly linked to more general psychiatric conditions. Major depressive disorder (MDD) is one of the most observed comorbidities among trauma-exposed individuals. Depressive symptoms may result from feelings of loss, helplessness, or self-blame associated with the traumatic experience [41]. Likewise, anxiety disorders including generalized anxiety, panic disorder, and social anxiety frequently occur following trauma, either as primary diagnoses or comorbid features [42]. Substance use disorders are also prevalent in trauma-exposed populations. Many individuals turn to alcohol or drugs as a form of self-medication to manage symptoms such as hyperarousal, insomnia, or emotional pain [43]. This pattern increases the risk of dependency and complicates treatment, particularly in populations such as veterans and survivors of childhood abuse.

Trauma exposure is also associated with personality disorders, particularly borderline personality disorder (BPD). Individuals with a history of early-life trauma or neglect may develop enduring patterns of emotional instability, impulsivity, and difficulties in relationships. While not all individuals with BPD have trauma histories, the association is strong enough that trauma-informed care is often recommended in treatment [44]. Another clinical consideration is the presence of dissociative symptoms, including depersonalization, derealization, or memory gaps. These symptoms often emerge in response to overwhelming trauma and can be transient or persistent. In some cases, they may form part of a dissociative subtype of PTSD or even meet criteria for a dissociative disorder [45].

Despite the use of categorical diagnoses like PTSD or depression, many researchers and clinicians are turning toward dimensional models of trauma-related psychopathology. The Research Domain Criteria (RDoC) framework, developed by the National Institute of Mental Health, emphasizes domains such as negative valence systems, arousal, and cognition rather than discrete diagnoses [46]. Similarly, the Hierarchical Taxonomy of Psychopathology (HiTOP) proposes that trauma-related symptoms are best understood as dimensions that cut across traditional diagnostic categories [47]. These models aim to capture the full range of responses to trauma and may offer more personalized treatment strategies.

It is also important to note that subthreshold symptoms can still result in significant impairment. Individuals may struggle with intrusive thoughts, irritability, social withdrawal, or somatic complaints, even if they do not qualify for a formal diagnosis. These symptoms can disrupt relationships, employment, and overall quality of life [48]. Additionally, trauma-related mental health problems often have delayed or fluctuating courses. Symptoms may emerge long after the traumatic event, especially when triggered by reminders or new stressors. In other cases, individuals may initially appear resilient but later develop symptoms during times of life transition or cumulative stress [49]. This variability underscores the importance of ongoing screening and flexible treatment approaches. Many trauma-related disorders also show significant overlap and comorbidity. For example, individuals with PTSD often meet criteria for depression or substance use disorders. This makes diagnosis and treatment more complex and highlights the need for integrated care models.

Finally, cultural factors play a critical role in how trauma symptoms are expressed and understood. Some individuals may present primarily with somatic symptoms, while others may frame their experiences in spiritual or existential terms. Clinicians must consider these variations to avoid misdiagnosis and ensure culturally sensitive care. In summary, the clinical consequences of trauma and chronic stress are wide-ranging and often multifaceted. Understanding this complexity is crucial for accurate diagnosis, appropriate treatment planning, and improving long-term outcomes.

## 5. Mediators and Moderators

**Figure 2:**
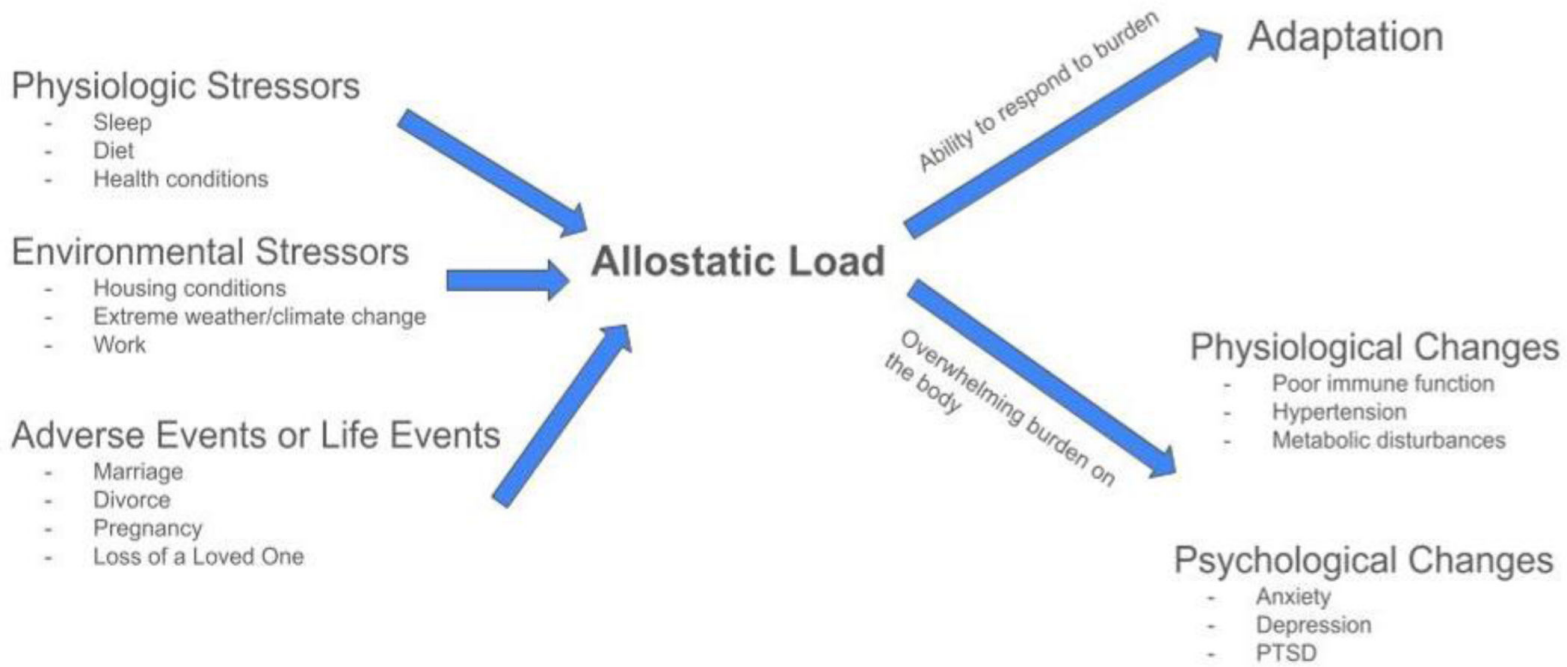
Examples of Physiological and environmental stressors, and adverse events that increase the allostatic load. Depending on the individual circumstances, this could result in a person’s ability to respond to burden leading to adaptation. However, the overwhelming burden may result in several physiological and psychological changes in the body leading to pathological conditions.

Not everyone who experiences trauma or chronic stress goes on to develop mental health problems. The variability in outcomes can often be explained by mediators and moderators. Understanding these elements helps clarify why some individuals develop psychiatric disorders while others show resilience or recover more quickly. One important mediating factor is the presence of daily stressors following the traumatic event. These are not necessarily related to the trauma itself but may include challenges such as financial strain, relationship conflicts, or caregiving burdens. Research suggests that daily stress can sustain or worsen trauma-related symptoms by keeping individuals in a heightened state of arousal or emotional exhaustion [50]. For example, someone recovering from a car accident may struggle more if they are also dealing with insurance problems, missed work, or limited social support. Daily stressors may also mediate the relationship between early trauma and later mental health problems (Figure 2). In this model, childhood trauma increases the likelihood of encountering stress in adulthood, which in turn contributes to depression, anxiety, or substance use disorders [51]. These secondary stressors can be both practical and emotional and often reflect the long-term consequences of disrupted development and relationships. Social support is one of the most widely studied moderators in trauma research. High levels of perceived emotional support from family, friends, or communities have been linked to reduced symptoms of PTSD and depression [52]. Conversely, a lack of support can increase psychological distress and complicate recovery. Support systems may provide a sense of safety, help individuals process emotions, and encourage treatment-seeking behaviors. Another key moderator is resilience, often defined as the ability to adapt or recover after adversity. Resilience is not a fixed trait but a dynamic process that can be influenced by personality, coping strategies, and environment. Individuals who demonstrate resilience may use more adaptive coping mechanisms, maintain hope for the future, or find meaning in their experiences (Figure 2). Programs that promote coping skills, emotional regulation, and problem-solving may enhance resilience and improve outcomes [53]. Contextual factors such as socioeconomic status, neighborhood safety, and access to healthcare also play a significant role. People living in unsafe or under-resourced environments may have fewer opportunities for recovery, more exposure to ongoing stress, and less access to mental health care. These structural inequities help explain why some populations experience higher rates of trauma-related disorders [54].

Cultural factors can also moderate how trauma is perceived and responded to. Some cultures emphasize collective coping or spirituality, which may protect against symptoms and promote community healing. Others may stigmatize emotional expression or mental health treatment, which could increase the risk for untreated distress [55]. Recent research has highlighted the interaction of these factors. For example, individuals with early trauma who face daily stress but have strong social support may fare better than those with similar trauma histories but poor support systems. This suggests that risk and protective factors do not operate in isolation; interventions should aim to strengthen protective factors while reducing ongoing stress [56]. Recognizing mediators and moderators is essential not only for understanding trauma outcomes but also for designing effective treatments. Tailoring interventions to account for social context, resilience capacity, and ongoing stressors can improve long-term recovery and reduce the burden of trauma-related conditions.

## 6. Population and Public Health Perspectives

While trauma is often conceptualized at the individual level, its effects frequently extend beyond the person and into communities and societies. Collective exposures such as natural disasters, pandemics, or wars can lead to widespread mental health consequences that require public health–level responses. Community disasters such as hurricanes, earthquakes, or terrorist attacks are associated with elevated rates of depression, anxiety, PTSD, and substance use disorders in affected populations [57]. These events often result in the loss of loved ones or property and cause prolonged uncertainty about the future. Research shows that the mental health effects of disasters are not evenly distributed. Individuals with fewer social and economic resources, those with preexisting mental health conditions, and members of marginalized communities tend to be more severely affected [58]. Mass trauma can overwhelm mental health systems and contribute to long-term psychiatric burdens. For example, populations affected by war often report high rates of PTSD and depression, sometimes years after the conflict has ended [59]. Children growing up in conflict zones are particularly vulnerable due to their developmental stage and continued exposure to insecurity [60]. Public health models of trauma emphasize the importance of population-level interventions that extend beyond individual treatment. The social-ecological framework, commonly used in injury prevention and violence reduction, has been adapted to address trauma and mental health (Figure 2). This model recognizes that mental health outcomes are shaped by interactions at multiple levels. These interactions include individual, relational, community, and societal; interventions must target each layer to be effective [61].

For example, individual-level strategies might focus on screening and early intervention, while community-level efforts could include safe housing initiatives, violence prevention programs, or school-based mental health services. At the policy level, trauma-informed legislation, funding for mental health infrastructure, and equitable healthcare access are essential for prevention and long-term recovery [62]. These multi-level approaches aim not only to treat mental illness but also to reduce risk factors and build resilience in populations. Screening and early intervention are cornerstones of trauma-informed public health efforts. Routine screening for trauma exposure in healthcare and educational settings has been shown to improve identification and referral for mental health support [63]. Tools such as the Adverse Childhood Experiences (ACEs) questionnaire are now commonly used in pediatric and primary care to identify individuals at risk for poor outcomes [64]. However, screening must be paired with access to trauma-informed services to be effective and ethical.

School-based mental health programs have been shown to reduce PTSD and anxiety symptoms in children exposed to trauma. These programs may include group therapy, psychoeducation, mindfulness training, and social-emotional learning [65]. School-based interventions often serve as a first line of support in post-disaster settings, particularly where formal mental health resources are limited.

On a broader scale, public mental health campaigns can help reduce stigma and increase awareness of trauma-related symptoms. These campaigns often use media messaging, peer support networks, and culturally tailored outreach to improve community engagement and resilience [66]. Importantly, public health approaches emphasize equity and access. Not all populations have the same risk of trauma exposure or the same ability to recover. People living in poverty, racial and ethnic minorities, LGBTQ+ individuals, and people with disabilities often face higher rates of trauma and more barriers to care [67]. Reducing these disparities is a central goal of trauma-informed public health. In summary, trauma is not just a clinical issue; it is a public health challenge. Addressing its widespread impact requires strategies that span from early screening to policy reform. By applying population-level models and targeting structural factors, public health efforts can prevent trauma-related mental illness and promote long-term mental wellness in communities.

## 7. Treatment and Prevention

Effective treatment and prevention of trauma-related mental health conditions requires a comprehensive approach that spans from individual to broader social and policy-level interventions. While not every trauma-exposed person develops a psychiatric disorder, many benefit from targeted interventions to reduce symptoms and prevent long-term complications.

At the individual clinical level, the most evidence-based treatments for trauma-related disorders are psychotherapeutic approaches, particularly those that involve trauma processing. Among these, trauma-focused cognitive behavioral therapy (TF-CBT) and eye movement desensitization and reprocessing (EMDR) have the strongest empirical support for reducing PTSD symptoms and associated distress [68]. These therapies aim to help individuals process traumatic memories, challenge maladaptive beliefs, and reduce physiological reactivity. Other validated approaches include prolonged exposure therapy, cognitive processing therapy, and narrative exposure therapy, each with varying levels of evidence across different populations [69].

Pharmacologic interventions are also used in the treatment of trauma-related disorders, although they are generally considered second-line or adjunctive treatments. Selective serotonin reuptake inhibitors (SSRIs), particularly sertraline and paroxetine, have been approved for the treatment of PTSD and may also address comorbid depression or anxiety [70]. Other medications, such as prazosin, have shown benefit in reducing trauma-related nightmares and sleep disturbances [71]. However, medication alone is usually less effective than psychotherapy, especially for addressing core trauma symptoms such as intrusive thoughts or emotional numbing [72]. Beyond symptom-focused treatment, prevention strategies are essential, particularly for individuals at high risk or in the early aftermath of trauma. Early intervention programs following disasters can help reduce the progression to full-blown PTSD in some individuals [73]. Psychological debriefing, once common, is now used more cautiously, as research has shown it may be ineffective or even harmful if implemented prematurely [57]. Community-based prevention efforts often focus on increasing awareness, reducing stigma, and promoting early access to services. These may include educational workshops, peer support groups, or public health campaigns aimed at helping individuals recognize trauma symptoms and seek help before symptoms become chronic [74]. In schools, trauma-informed programs that teach coping skills, emotional regulation, and mindfulness have been associated with reduced behavioral problems and improved mental health in children exposed to adversity [75].

At a policy level, trauma prevention involves addressing the upstream conditions that increase vulnerability to trauma and stress. This includes reducing poverty, preventing violence, supporting safe housing, and ensuring access to equitable healthcare. Policies that address adverse childhood experiences (ACEs) can reduce the population-level burden of trauma-related illness over time [76]. Recent research has also focused on biomarkers and personalized approaches to treatment. Biomarkers such as cortisol levels and inflammatory markers, or neuroimaging findings are being explored to help predict who may benefit from specific treatments or to monitor recovery over time [77]. While not yet ready for routine clinical use, these tools represent a promising direction in tailoring interventions to individual needs. Digital and technological tools are another emerging area in trauma care. Mobile apps, teletherapy, and internet-based CBT programs have shown preliminary success in expanding access to trauma-focused treatment, particularly in underserved or remote populations [78]. These approaches are especially relevant in the context of global crises, such as pandemics or natural disasters, where in-person care may be disrupted. In summary, treatment and prevention of trauma-related conditions require a layered approach that combines clinical expertise with community engagement and systemic change. By integrating psychotherapy, pharmacology, early intervention, and policy reform, mental health systems can more effectively respond to the diverse needs of trauma survivors and reduce the long-term burden of trauma on individuals and society.

## 8. Future Directions and Research Gaps

Although significant advances have been made in understanding and treating trauma-related mental health conditions, important gaps remain in research, clinical practice, and public health responses. Addressing these gaps will require a continued focus on refining definitions, improving diagnostic frameworks, and expanding research to better capture diverse experiences and populations. One ongoing challenge is the lack of consensus on definitions and diagnostic criteria for trauma-related conditions. While PTSD is well-established in both the DSM-5 and ICD-11, there is ongoing debate about the inclusion and boundaries of related diagnoses such as complex PTSD, prolonged grief disorder, and adjustment disorder [79]. These distinctions are important because they affect who qualifies for treatment, what types of interventions are offered, and how outcomes are measured in research. There is also a need for more longitudinal research that tracks individuals over time, particularly from childhood into adulthood. Most trauma research remains cross-sectional, making it difficult to determine causal relationships and long-term trajectories [80]. Understanding how early experiences influence later mental health and how resilience develops requires sustained observation and more detailed developmental models.

Mechanistic studies are another priority. Although progress has been made in identifying biomarkers, brain changes, and epigenetic mechanisms, many of these findings are still preliminary [81]. Future work should aim to clarify the biological pathways linking trauma to mental illness and identify which of these can be targeted through treatment or prevention. Integrating findings from neuroscience, genetics, and immunology will be essential for moving toward personalized interventions.

Another area of growth is inclusive and culturally sensitive research. Many studies have focused on western or high-income populations, leaving major gaps in understanding how trauma manifests and is treated in more diverse contexts [82]. Future research must address the needs of marginalized groups, including refugees, Indigenous populations, LGBTQ+ individuals, and people living in low-resource settings. Finally, more intervention studies are needed, particularly those that go beyond symptom reduction to measure functional recovery, quality of life, and long-term wellbeing. Innovations in digital health, group-based care, and trauma-informed systems should be rigorously evaluated across different settings. Closing these research gaps will allow for more precise, equitable, and effective responses to trauma.

## Conclusion

Trauma and chronic stress are powerful determinants of mental health, shaping emotional, cognitive, and behavioral outcomes across the lifespan. From isolated traumatic events to long-term exposure to daily adversity, these experiences can lead to a wide range of psychiatric conditions. However, not all individuals respond to trauma in the same way, and outcomes are influenced by an interplay of biological, psychological, and social factors.

At the biological level, research has revealed that trauma affects stress regulation systems, brain structure and function, immune responses, and gene expression. These changes may persist long after the trauma has passed and help explain why some individuals are more vulnerable to mental illness. At the clinical level, diagnostic models are evolving to better capture the complexity and variability of trauma-related symptoms, including the move toward dimensional approaches and greater recognition of overlapping and comorbid presentations. Equally important are the social and environmental contexts in which trauma occurs. Poverty, inequality, lack of access to care, and limited social support all increase the risk of poor mental health outcomes and reduce the likelihood of recovery. Public health models have begun to address these broader influences by promoting trauma-informed policies, early intervention, and community-based care. These efforts aim not only to reduce symptoms but also to address the root causes of vulnerability. The field is also beginning to shift toward a more hopeful and empowering view of trauma recovery. While much of the focus has been on pathology, growing attention is being paid to post-traumatic growth and the capacity for healing. Recognizing and supporting individual and community strengths can play a crucial role in recovery and long-term wellbeing. In conclusion, trauma and stress are not isolated clinical issues but multidimensional public health challenges. Addressing them requires an integrated, multi-level approach that spans from neuroscience to social justice. Continued investment in research, prevention, and equitable care systems will be essential to reducing the burden of trauma-related mental illness and fostering resilience in individuals and communities.

## Key Points, Challenges, and Outstanding Questions

- Acute trauma (e.g., violence, disasters) can lead to post-traumatic stress disorder, and chronic stress (due to poverty, caregiving, discrimination, and others) is associated with slower, cumulative effective effects leading to anxiety, depression, substance use disorder, panic disorder, and other cumulative psychiatric disorders.
- The mental health symptoms can range from flashbacks, nightmares, and avoidance to hypervigilance, concentration problems, sleep disturbances, emotional numbness, impacting relationships and overall quality of life.
- Trauma and stressors differ in intensity and duration, but both significantly impact mental health. A personal or family history of mental illness or substance use can increase vulnerability, and lack of social support from family and friends can increase the severity of symptoms.
- Exposure to trauma and stress is common globally but not evenly distributed.
- Vulnerable populations such as refugees, children, and those facing socioeconomic hardship experience disproportionate exposure, compounding mental health risks.
- Epidemiological data show a dose-response relationship between adversity and mental illness. The number and types of adverse experiences are strongly correlated with the severity and persistence of psychiatric conditions, including PTSD, depression, and substance use.
- Trauma alters neurobiological systems involved in stress regulation, memory, and emotion. Dysregulation of the HPA axis, reduced hippocampal volume, heightened amygdala reactivity, and impaired prefrontal control contribute to psychopathology.
- Early life stress is biologically embedded and may have intergenerational effects.
- Epigenetic modifications and gene-environment interactions can influence both immediate and long-term mental health, with implications for future generations.
- Diagnostic challenges arise from overlapping symptoms and comorbidities.
- Traditional categorical models often fall short in capturing the complexity of trauma-related disorders, prompting interest in dimensional frameworks like RDoC and HiTOP.
- Social support and resilience are crucial protective factors.
- Community context, access to care, interpersonal relationships, and safe and non-judgmental space have powerful impact on individual outcomes and recovery potential.
- Evidence-based treatments are available but not equally accessible.
- Trauma-focused therapies and pharmacologic interventions show efficacy, but implementation is limited by barriers such as stigma, provider availability, and systemic inequities.
- Public health approaches are essential for prevention and intervention.
- Trauma-informed care, school-based programs, and policy-level reforms offer scalable solutions but require coordination across sectors and sustained investment.
- Future directions include identifying biomarkers, refining diagnostic criteria, and addressing social determinants. Major challenges remain in translating biological findings into clinical tools, designing inclusive longitudinal studies, and developing personalized and equitable care strategies.
